# Human papillomavirus, p16 and p53 expression associated with survival of head and neck cancer

**DOI:** 10.1186/1750-9378-5-4

**Published:** 2010-02-11

**Authors:** Elaine M Smith, Linda M Rubenstein, Henry Hoffman, Thomas H Haugen, Lubomir P Turek

**Affiliations:** 1 Department of Epidemiology, College of Public Health, University of Iowa, Iowa City, IA 52242, USA; 2 Department of Otolaryngology, College of Medicine, University of Iowa, Iowa City, IA 52242, USA; 3 Veterans Affairs Medical Center, and Department of Pathology, College of Medicine, University of Iowa, Iowa City, IA 52242, USA

## Abstract

**Background:**

P16 and p53 protein expression, and high-risk human papillomavirus (HPV-HR) types have been associated with survival in head and neck cancer (HNC). Evidence suggests that multiple molecular pathways need to be targeted to improve the poor prognosis of HNC. This study examined the individual and joint effects of tumor markers for differences in predicting HNC survival. P16 and p53 expression were detected from formalin-fixed, paraffin-embedded tissues by immunohistochemical staining. HPV DNA was detected by PCR and DNA sequencing in 237 histologically confirmed HNC patients.

**Results:**

Overexpression of p16 (p16+) and p53 (p53+) occurred in 38% and 48% of HNC tumors, respectively. HPV-HR was detected in 28% of tumors. Worse prognosis was found in tumors that were p53+ (disease-specific mortality: adjusted hazard ratios, HR = 1.9, 95% CI: 1.04-3.4) or HPV- (overall survival: adj. HR = 2.1, 1.1-4.3) but no association in survival was found by p16 status. Compared to the molecular marker group with the best prognosis (p16+/p53-/HPV-HR: referent), the p16-/p53+/HPV- group had the lowest overall survival (84% vs. 60%, p < 0.01; HR = 4.1, 1.7-9.9) and disease-specific survival (86% vs. 66%, p < 0.01; HR = 4.0, 1.5-10.7). Compared to the referent, the HRs of the other six joint biomarker groups ranged from 1.6-3.4 for overall mortality and 0.9-3.9 for disease-specific mortality.

**Conclusion:**

The p16/p53/HPV joint groups showed greater distinction in clinical outcomes compared to results based on the individual biomarkers alone. This finding suggests that assessing multiple molecular markers in HNC patients will better predict the diverse outcomes and potentially the type of treatment targeted to those markers.

## Background

Infection with human papillomavirus high risk (HPV-HR) types is causally related to the development of HNC independent of tobacco and alcohol use [1-3]. HPV infection has been demonstrated to play a role in the molecular pathways through its viral oncoproteins, E6 and E7. These proteins increase degradation of p53 and interfere with pRb function leading to upregulation of p16^INK4a ^by loss of negative feedback control [4]. P16^INK4a ^and TP53 are tumor suppressor genes and key targets in the loss of cell cycle control [5]. Overexpression of p16 (p16+) in HPV-HR infection has been demonstrated in a high percentage of cervical and HNC cancers [5-7] and it has been suggested that p16^INK4a ^expression may serve as a surrogate biomarker of oncogenic HPV infection in predicting HPV-related tumors [8,9]. Better prognosis has been shown in HPV-HR [10] and in p16+ HNC tumors [7,9,11]. However, the signals and pathways that determine p16 expression in humans [12] or upregulation in HPV-related tumors are not well understood. Although p53 serves an important role in carcinogenesis, it is a controversial prognostic indicator with some studies showing p53 expression linked to decreased HNC survival [7,8,13] and others finding no correlation [12,14,15]. TP53 wild type has been shown to be highly correlated with HPV infection in HNC whereas TP53 mutations are rare in infected tumors [16,17]. A large investigation [16] found that HPV HNC tumors had higher overall survival after adjusting for the effect of TP53 mutation. We have shown that disease-specific and recurrence-free survival were highest in the joint biomarkers p16+/HPV-HR and p53-/HPV-HR HNC tumors, and lowest in p16-/HPV- and p53+/HPV- tumors, and survival was intermediate but different among the other joint p16/HPV and p53/HPV biomarker groups, suggesting that prognostic outcomes of HNC based on multiple biomarkers need to be evaluated jointly to more accurately determine clinical outcomes and possibly alternative, targeted treatment regimens [7,13].

Although some studies have examined the association between two joint markers, p16, p53, or HPV, in HNC [7,8,12,16,18], few have evaluated their joint effects on survival. None has evaluated the additional clinical outcome that evaluating three biomarker tests might provide, a common practice in medicine to perform multiple different diagnostic assessments. Previous investigations have adjusted one marker to examine the risks and outcomes associated with another marker of interest; however, because of the potential differences in the molecular pathways leading to tumorigenesis, the joint effects of these genes need to be evaluated. By joint effect we are referring to an examination of the combined markers, p16, p53, and HPV, as eight distinct tumor groups with the potential for as many different clinical outcomes; and with different effects associated with other risk factors and confounders (e.g., tobacco, stage). Among those that have attempted to examine joint effects, none has examined multiple markers adjusting for effects of age, stage, tobacco or alcohol use [18,19]. Thus, the prognostic significance associated with alterations in multiple molecular markers remains unclear. This investigation presents a large group of newly diagnosed HNC cases examined for the association by joint p16, p53, and HPV-HR status in HNC tumors to evaluate their effects on survival.

## Results

### Risk Factors and Pathologic Characteristics by p16, p53 and HPV Status

Among the 237 HNC cases, 61% were male and the average patient age was 60 years. P16 (p16+) was overexpressed in 38%, p53 (p53+) was overexpressed in 48%, and HPV-HR was detected in 28% of HNC tumors. When examined by site, 68% of the oropharyngeal, 28% of the oral cavity, and 16% of the laryngeal cancers were p16+; p53+ prevalence was 47% in the oropharynx, 46% in the oral cavity, and 59% in the larynx; and 63% of the oropharynx, 15% of the oral cavity, and 9% of the larynx tumors were HPV-HR positive. There were three HPV-HR types detected: 94% were HPV-16, 5% HPV-33, and 1% HPV-18.

Table 1 shows the association and p-values between p16, p53, and HPV outcome status and potential risk factors and pathologic characteristics of HNC, first for all HNC histologic types and separately for SCC. Reverence to protein overexpression or positive infection status is noted by the "+" symbol and lack of expression or HPV negative for infection by the "-" symbol. After adjusting for age, alcohol, tobacco and the other biomarkers, p16+ was associated with a significantly reduced risk of p53 expression (OR = 0.5, 95% CI: 0.2-0.9) and a higher risk of in HPV-HR detection in tumors (OR = 16.1, 7.6-33.9). P53+ showed an inverse relationship with p16+ but was not significantly related to patient characteristics or HPV status. In addition to the significant association with p16+ status, HPV-HR was associated with alcohol (≥ 1-21 drinks/week: OR = 2.4, 0.9-6.0, >21 drinks/week: OR = 2.7, 0.9-7.9) and tobacco use (>0-30 pack-years: OR = 6.4, 2.1-19.3). P16 overexpression and HPV-HR were significantly higher in oropharyngeal (OR = 4.0, 1.1-13.8, OR = 8.4, 1.8-38.2 respectively) but not in oral cavity compared to laryngeal tumors. Later stage (IV, OR = 2.6, 1.2-5.7) and higher grade (OR = 2.0, 0.97-4.3) were more likely to be detected in p16+, and positive nodal involvement was more frequently found in p16+ (OR = 2.1, 1.05-4.0) and HPV-HR (OR = 2.1, 0.99-4.4) cancers. Tumor site and pathologic characteristics were not related to p53 status. Table 1 shows that nonSCC cases were more likely than SCC cases to be p16+ (55% vs. 36%) but less likely to be HPV-HR (14% vs. 29%,). Findings limited to SCC showed greater risk of HPV-HR among males (p = 0.05), moderate and heavy alcohol-related users (≥ 1-21: p = 0.002; >21: p = 0.02), oropharynx (p = 0.008); and greater risk of p16+ in poor/undifferentiated tumors (p = 0.02).

**Table 1:** Risk Factors and Pathologic Characteristics of HNC by p16, p53, and HPV Status for All Histologic and SCC^1 ^Histologic Types

Characteristics	p16-	p16+	All/SCC	p53+	p53-	All/SCC	HPV-	HPV-HR	All/SCC
	N = 148 (%)	N = 89 (%)	p-value^2^	N = 114 (%)	N = 123 (%)	p-value^2^	N = 171 (%)	N = 66 (%)	p-value^2^
Gender									
Female	64 (43.2)	28 (31.5)		49 (43.0)	43 (35.0)		75 (43.9)	17 (25.8)	
Male	84 (56.8)	61 (68.5)	0.5/0.7	65 (57.0)	80 (65.0)	0.5	96 (56.1)	49 (74.2)	0.1/0.05
Age Group									
≤ 55 years	48 (32.4)	41 (46.1)	0.2/0.2	37 (32.5)	52 (42.3)	0.4	58 (33.9)	31 (47.0)	0.6/0.4
>55 years	100 (67.6)	48 (53.9)		77 (67.5)	71 (57.7)		113 (66.1)	35 (53.0)	
Alcohol									
Never	45 (30.6)	22 (24.7)		31 (27.5)	36 (29.2)		55 (32.4)	12 (18.2)	
≥ 1-21	58 (39.5)	39 (43.8)	0.9/0.2	45 (39.8)	52 (42.3)	0.6	66 (38.8)	31 (47.0)	0.06/0.002
>21	44 (29.9)	28 (31.5)	0.8/0.4	37 (32.7)	35 (28.5)	0.3	49 (28.8)	23 (34.8)	0.08/0.02
Tobacco									
Never	34 (23.1)	19 (21.4)		26 (22.8)	27 (22.5)		45 (26.4)	8 (12.1)	
>0-30	38 (25.9)	27 (30.3)	0.2/0.4	32 (28.1)	33 (27.0)	0.7	37 (21.8)	28 (42.4)	0.001/0.007
>30	75 (51.0)	43 (48.3)	0.9/0.3	56 (49.1)	62 (50.8)	0.97	88 (51.8)	30 (45.5)	0.3/0.8
p16 Status									
Positive	-	-	-	32 (28.1)	57 (46.3)		36 (21.1)	53 (80.3)	< 0.0001/ < 0.0001
Negative	-	-	-	82 (71.9)	66 (53.7)	0.03	135 (78.9)	13 (19.7)	
p53 Status									
Positive	82 (55.4)	32 (36.0)	0.03/0.02	-	-	-	88 (51.5)	26 (39.4)	0.99/0.9
Negative	66 (44.6)	57 (64.0)		-	-	-	83 (48.5)	40 (60.6)	
HPV Status									
HR	13 (08.8)	53 (59.6)		26 (22.8)	40 (32.5)	0.98	-	-	-
Negative^3^	135 (91.2)	36 (40.4)	< .0001/< 0.001	88 (77.2)	83 (67.5)		-	-	-
Site									
Oropharynx	22 (14.9)	46 (51.7)	0.03/0.05	32 (28.1)	36 (29.3)	0.8	25 (14.6)	43 (65.2)	0.01/0.008
Oral Cavity	99 (66.9)	38 (42.7)	0.3/0.4	63 (55.)	74 (60.1)	0.4	117 (68.4)	20 (30.3)	0.99/0.97
Larynx/Hypo^4^	27 (18.2)	5 (5.6)		19 (16.7)	13 (10.6)		29 (17.0)	3 (4.5	
Stage									
0/I/II	56 (38.1)	14 (15.7)		35 (30.7)	35 (28.7)		60 (35.3)	10 (15.2)	
III	25 (17.0)	17 (19.1)	0.2/0.2	20 (17.5)	22 (18.0)	0.6	28 (16.5)	14 (21.2)	0.2/0.4
IV	66 (44.9)	58 (65.2)	0.02/0.01	59 (51.8)	65 (53.3)	0.5	82 (48.2)	42 (63.6)	0.2/0.6
Tumor Grade									
Well/Mod	118 (81.4)	55 (64.0)		87 (77.7)	86 (72.3)		132 (78.6)	41 (65.1)	
Poor/Undif^5^	27 (18.6)	31 (36.0)	0.06/0.02	25 (22.3)	33 (27.7)	0.7	36 (21.4)	22 (34.9)	0.6/0.7
Nodal Involvement									
Yes	59 (40.1)	61 (68.5)	0.04/0.04	53 (46.9)	67 (54.5)	0.9	73 (42.9)	47 (71.2)	0.05/0.2
No	88 (59.9)	28 (31.5)		60 (53.1)	56 (45.5)		97 (57.1)	19 (28.8)	
Histology									
SCC	138 (93.2)	77 (86.5)	0.08	106 (93.0)	109 (88.6)	0.2	152 (88.9)	63 95.4)	0.1
nonSCC	10 (6.8)	12 (13.5)		8 (7.0)	14 (11.4)		19 (11.1)	3 (4.6)	

^1 ^SCC = squamous cell carcinoma; ^2^The left side of slash p-value is in reference to All histologies and the right side p-value refers to SCC only; the p-value is in reference to positive vs. negative biomarker status for each risk factor, p-values are adjusted for age, alcohol, tobacco, p16, p53, and HPV; ^3 ^HPV negative (-) for all low-risk and high-risk HPV types; ^4 ^Hypo = Hypopharynx; ^5 ^Undif = Undifferentiated.

The eight p16/p53/HPV joint biomarker groups also were compared for potential differences in risk factors, adjusting for age, tobacco, and alcohol use with each group compared to the p16+/p53-/HPV-HR group (data not shown). This group was chosen as the referent on the basis of our previous research [7,10,13] and the findings in this study regarding the biomarker group with the best survival outcome. All analyses examined both SCC separately and all histologic groups, with no differences in findings. The two biomarker groups that were both p16- and HPV- were significantly less likely to include males: p16-/p53+/HPV- and p16-/p53-/HPV- (p = 0.04-0.01). Moderate use of tobacco (>0 ≤ 30 pack-years) was more frequent in the referent (41%) than in the three groups that were HPV- (p16+/p53-/HPV- 10%, p16-/p53-/HPV- 25%, and p16+/p53+/HPV- 13%; p = 0.07-0.02); heavy tobacco use (>30 pack-years) also was less frequently reported in the p16+p53+HPV- group (38% versus 49%, p = 0.03). Assessment of tumor characteristics indicated that all joint groups, except p16+/p53+/HPV-HR, were less likely to have later stage (p = 0.03 < 0.002) and nodal involvement (p = 0.01-<0001) compared to the referent. These findings are similar to the individual biomarkers shown in table 1. Except for p16+/p53+/HPV-HR, all joint groups were less likely to be detected in the oropharynx (p = 0.02 < 0.0001) compared to the referent.

### Survival

There were 237 cases with overall survival data, 234 cases with disease-specific survival (3 cases could not be classified as free of disease), and 203 cases with data on recurrence-free survival (31 cases were never disease-free). The median time to death or last follow-up for the HNC cases was 1.9 years (range 0.03-10.1 years) for overall survival and 1.8 years for disease-specific survival (range: 0.03-10.1 years). There were 73 deaths in the overall survival, 51 in disease-specific survival, and 46 with recurrence.

Table 2 shows the median survival for cases based on the individual and joint biomarker groups and for all histologies combined and SCC separately. Those who had tumors that were either p16+ or HPV-HR had significantly higher median years of overall, disease-specific, and recurrence-free survival, whereas p53 status was unrelated to survival time. When we compared SCC to nonSCC (data not shown), nonSCC cases had longer median years of disease-specific survival in the p53+ (2.8 years vs. 1.7 years) and HPV-HR groups (2.9 vs. 2.3); and had longer median years of recurrence-free survival for individual biomarker comparisons (p16-: 2.3 vs. 1.4; p53-: 2.7 vs. 1.9; HPV-HR: 3.1 vs. 2.3; HPV-: 2.2 vs. 1.4).

**Table 2:** Frequency and Median Years of Overall, Disease-Specific and Recurrence-free Survival by p16/p53/HPV Status

			Overall Survival				Disease-specific Survival				Recurrence-free Survival (N = 203)			
			
			All Histologies N = 237		SCC N = 215		All Histologies N = 234		SCC N = 212		All Histologies N = 203		SCC N = 185	
**p16/p53/HPV Status**	**N^1^**	**SCC (N)^1^**	**Median**	**p-value^2^**	**Median**	**p-value^2^**	**Median**	**p-value^2^**	**Median**	**p-value^2^**	**Median**	**p-value^2^**	**Median**	**p-value^2^**

p16+	89	77	2.3	referent	2.3	referent	2.3	referent	2.3	referent	2.3	referent	2.3	referent
p16-	148	138	1.7	0.009	1.7	0.01	1.7	0.008	1.7	0.01	1.4	0.0001	1.4	< 0.001
p53-	123	109	2.0	referent	2.0	referent	1.9	referent	1.9	referent	1.9	referent	1.9	referent
p53+	114	106	1.7	0.39	1.7	0.24	1.7	0.41	1.7	0.25	1.6	0.17	1.5	0.15
HPV-HR	66	63	2.3	referent	2.3	referent	2.3	referent	2.3	referent	2.5	referent	2.3	referent
HPV-^2^	171	152	1.7	0.002	1.6	0.001	1.6	0.002	1.6	0.001	1.5	0.0008	1.4	< 0.001
p16+/p53-/HPV-HR	37	36	2.3	referent	2.3	referent	2.3	referent	2.3	referent	2.7	referent	2.6	referent
p16-/p53-/HPV-HR	3	2	2.8	0.77	3.1	0.46	2.8	0.77	3.1	0.46	1.9	0.50	1.9	0.54
p16+/p53+/HPV-HR	16	15	2.8	0.93	2.8	0.93	2.8	0.93	2.8	0.93	2.7	0.48	2.5	0.48
p16-/p53+/HPV-HR	10	10	1.3	0.07	1.3	0.09	1.3	0.07	1.3	0.09	1.0	0.02	1.0	0.03
p16-/p53+/HPV-	72	69	1.6	0.004	1.6	0.006	1.6	0.003	1.6	0.006	1.4	0.0001	1.4	< 0.001
p16+/p53-/HPV-	20	14	1.2	0.01	1.1	0.07	1.2	0.01	1.1	0.07	1.2	0.008	0.9	0.05
p16-/p53-/HPV-	63	57	1.8	0.02	1.8	0.01	1.8	0.02	1.7	0.01	1.5	0.001	1.4	< 0.001
p16+/p53+/HPV-	16	12	2.4	0.47	1.3	0.22	2.4	0.47	1.3	0.22	2.7	0.68	1.9	0.35

^1^Number of cases with survival data; ^2 ^p-value for differences in median years within biomarker status, ^3 ^HPV- for all low-risk and high-risk HPV types.

More survival distinctions were apparent in the joint marker analyses. Median survival associated with the three survival outcomes were near or significantly longer in the referent group (p16+/p53-/HPV-HR) compared to four other marker groups, all of which were p16- and/or HPV- (p16-/p53+/HPV-HR, p16-/p53+/HPV-, p16+/p53-/HPV-, and p16-/p53-/HPV-). The other three joint biomarker groups (p16-/p53-/HPV-HR, p16+/p53+/HPV-HR, and p16+/p53+/HPV-) had similar 2-year overall, disease-specific, and recurrence-free survival to that of the referent. These three groups were p16+ and/or HPV-HR, thus having the opposite marker status to that of the worse median survival groups. When analyses were limited to SCC, results were similar (table 2), although the p16+/p53+/HPV- group reported nonstatistically significantly worse median survival than the combined histologic group (table 2).

Table 3 presents the adjusted hazard ratios for overall survival and disease-specific survival of HNC associated with the three biomarkers, individually and jointly, for all histologic types and SCC. Because of the reduced sample size to assess recurrence-free analyses by the joint groups, power was low and data were not presented in the table. Multivariate analysis demonstrated that p16, p53, HPV, age, and stage were independently associated with survival and thus were included in the adjustments whereas alcohol, tobacco, tumor site, and treatment were not independently significantly associated with survival and were excluded. For all histologies, tumors that were p16- had only slightly worse disease-specific survival compared to p16+ tumors; those that were p53+ had significantly worse disease-specific survival for all histologies and SCC patients only; and HPV- cases had significantly worse overall survival and elevated disease-specific mortality for combined and SCC histologies. Recurrence-free survival for all histologies (data not shown) was worse among those who were p16- (HR = 2.9, 1.2-6.9), or p53+ (HR = 2.3, 1.2-4.4) whereas HPV was not related to recurrence (HR = 1.0, 0.4-2.4). For SCC only, recurrence free survival was elevated among those who were p16- (HR = 2.1, 0.8-5.4), worse for p53+ (2.1, 1.1-4.6) and not related to HPV (HR = 1.3, 0.5-3.2).

**Table 3:** Adjusted^1 ^Hazard Ratio for Overall and Disease-specific Survival

Risk Factors	Overall Survival		Disease-Specific Survival	
	HR (95% CI)		HR (95% CI)	
	**All Histologies** **N = 237**	**SCC** **N = 215**	**All Histologies** **N = 234**	**SCC** **N = 212**

p16 Status				
p16-	1.2 (0.7-2.2)^1^	0.9 (0.5-1.7)	1.5 (0.7-3.1)^1^	1.0 (0.5-2.2)
p16+	referent	referent	referent	referent
				
p53 Status				
p53-	referent	referent	referent	referent
p53+	1.4 (0.9-2.2)^1^	1.3 (0.8-2.2)	1.9 (1.04-3.4)^1^	1.9 (1.01-3.5)
				
HPV Status				
HPV-HR	referent	referent	referent	referent
HPV-	2.1 (1.1-4.3)^1^	2.6 (1.2-5.3)	1.6 (0.7-3.7)^1^	2.1 (0.9-4.8)
				
p16/p53/HPV Status^3^				
p16+/p53-/HPV-HR	referent	referent	referent	referent
p16-/p53-/HPV-HR	NE^3^	NE^3^	NE^3^	NE^3^
p16+/p53+/HPV-HR	1.6 (0.4-5.7)	1.8 (0.5-6.3)	0.9 (0.2-4.7)	1.0 (0.2-5.3)
p16-/p53+/HPV-HR	3.2 (0.8-13.4)	2.8 (0.7-11.5)	3.9 (0.9-16.9)	3.4 (0.8-14.9)
p16-/p53+/HPV-	4.1 (1.7-9.9)	3.7 (1.5-9.0)	4.0 (1.5-10.7)	3.7 (1.4-9.9)
p16+/p53-/HPV-	2.9 (0.9-9.1)	3.6 (1.1-11.1)	1.7 (0.4-7.3)	2.2 (0.5-9.2)
p16-/p53-/HPV-	3.1 (1.2-7.9)	3.2 (1.2-8.2)	1.9 (0.6-5.7)	1.9 (0.6-5.9)
p16+/p53+/HPV-	3.4 (1.1-10.3)	4.6 (1.4-14.5)	2.9 (0.8-10.3)	4.6 (1.3-16.1)
				
Age^2^	1.03 (1.01-1.05)	1.04 (1.02-1.1)	1.03 (1.01-1.05)	1.04 (1.01-1.1)
				
Stage				
I/II	referent	referent	referent	referent
III/IV	3.1 (1.7-5.9)	3.1 (1.6-6.0)	4.6 (1.9-10.9)	4.5 (1.9-11.0)

^1^Adjusted for p16, p53, HPV, age, and stage; ^2^NE = not estimable due small sample size

**Figure 1:**
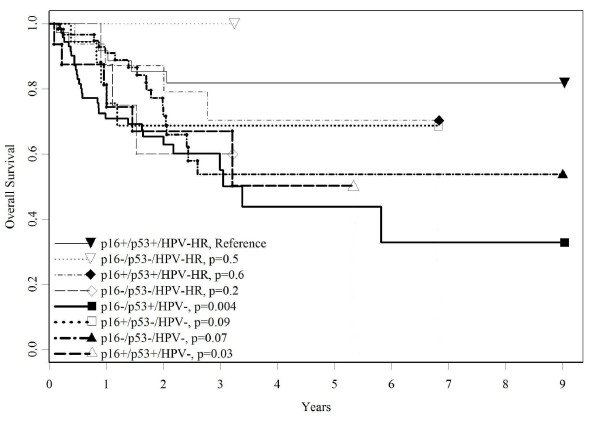
**Overall survival by p16, p53, and HPV status**. Survival curves based on the Kaplan-Meier method. Significance was based on Log-rank comparisons of each group to the reference group, p16+/p53-/HPV-HR.

**Figure 2:**
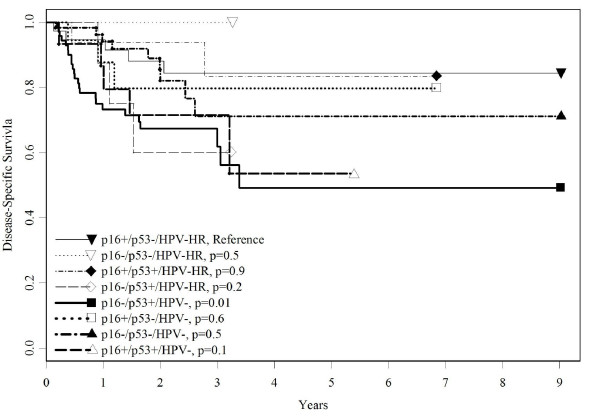
**Disease-specific survival by p16, p53, and HPV status**. Survival curves based on the Kaplan-Meier method. Significance was based on Log-rank comparisons of each group to the reference group, p16+/p53-/HPV-HR.

**Figure 3:**
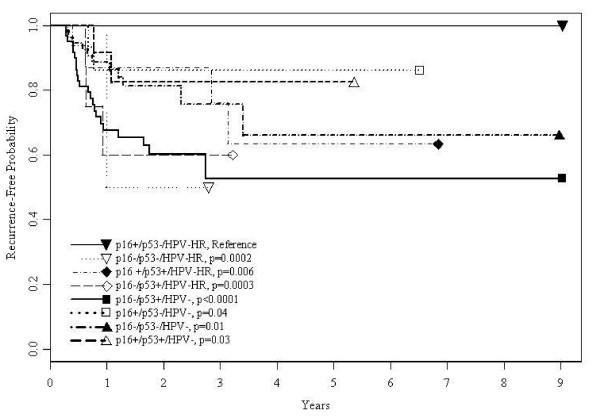
**Recurrence-free survival by p16, p53, and HPV status**. Survival curves based on the Kaplan-Meier method. Significance was based on Log-rank comparisons of each group to the reference group, p16+/p53-/HPV-HR.

Next the biomarkers were evaluated as joint groups for prognosis (table 3). In contrast to the referent (p16+/p53-/HPV-HR), the p16-/p53+/HPV- group had the lowest overall survival (adj. HR = 4.1, 84% vs. 60%, p < 0.01), disease-specific survival (HR = 4.0; 86% vs. 66%, p < 0.01), and recurrence-free survival (HR = 19.7, 50% versus 0%, p < 0.0001; data not shown). Overall survival was worse among all groups that had HPV- status (table 3 and figure 1) whereas the same was true for disease-specific survival in three of the p53+ groups: p16-/p53+/HPV-HR, p16-/p53+/HPV-, p16+/p53+/HPV- (table 3 and figure 2). Two other joint groups, p16+/p53-/HPV- and p16-/p53-/HPV-, had elevated but not statistically significant worse disease-specific survival risks, likely due to the more limited number of cases in those groups. For combined histologies, all joint groups except p16+/p53-/HPV- and p16+/p53+/HPV- had significantly higher risk of recurrence compared to the referent group (HRs = 8.6-19.7, p = 0.04- < 0.004; figure 3) although CIs were wide (data not shown in table). The HRs for p16+ p53-/HPV- and p16+/p53+/HPV- also were elevated (5.9, 0.5-66.9, p = 0.15; 5.5, 0.5-64.0, p = 0.17, respectively). Table 3 also shows that when SCC cases were examined, only the p16+/p53+/HPV- group showed worse HRs, compared to the joint histologies, for overall (4.6 vs. 3.3), disease-specific (4.6 vs. 2.9), and recurrence-free survival HR (9.1 vs. 5.5, 17% vs. 12%, data not shown), respectively. For SCC cases, p16-/p53+/HPV-, had the highest risk of recurrence (HR = 19.7, CI: 2.5-152.2, p-value = .004)) followed by p16-/p53+/HPV-HR (HR = 16.6, CI: 2.1-130.1, p-value = 0.004).

Other risk factors for survival included age which showed a small increased risk per year with prognostic outcomes (table 3). Patients with stages III/IV disease had significantly worse overall survival and even worse disease-specific survival but not recurrence (HR = 1.3, 0.7-2.5) compared to earlier stage tumors. Two-way interactions between the biomarkers showed no significant effect modification between p16, p53 and HPV for any of the clinical outcomes (p > 0.10, data not shown). The 3-way interaction among the biomarkers could not be examined for survival because there were too few deaths among the HPV-HR group whereas the 3-way interaction for recurrence showed a near significant interaction effect (p > 0.05- < 0.1). None of the tests for additive interactions was statistically significant.

We examined two-year survival rates (data not shown) for comparison with the survival analyses (table 3). Only the p16-/p53+/HPV- group had significantly worse adjusted (age, stage) two-year overall (83% versus 54%, adj. p = 0.003) and disease-specific survival (86% vs. 54%, adj. p = 0.002) compared to the referent group. One other joint molecular group (p16+/p53+/HPV-HR) had similar overall and disease-specific survival to the referent while the other joint groups had nonsignificantly lower two-year survival (data not shown). There were no recurrences (0%) at two years in the referent group, p16+/p53-/HPV-HR, whereas recurrence for the same period ranged between 17% and 51% in the other joint marker groups: p16-/p53+/HPV- (51%, p = 0.0001), adj. p16-/p53-/HPV- (30%, adj. p = 0.004), p16+/p53-/HPV- group (25%, p = 0.06), p16+/p53+/HPV- (20%, p = 0.09), and p16+/p53+/HPV-HR (17%, p = 0.12). The p16-/p53-/HPV-HR and p16-/p53+/HPV-HR groups had inadequate numbers of cases to evaluate two-year recurrence. There were no major differences in survival when histology was limited to SCC.

Since effectiveness of initial treatment may be influenced by the status of the molecular markers, patients were examined for disease-specific survival by therapy (data not shown). Compared to the oropharynx, cancers of the oral cavity were more likely to receive surgery (48% vs. 12%) or surgery/XRT (44% vs. 26%) and less likely to be treated by XRT (2% vs. 28%) or XRT/chemotherapy (1% vs. 25%). Because of small numbers, analyses could not be examined by site and could only be calculated for a few joint biomarker groups. Compared to the referent (p16+/p53-/HPV-HR), the worst survival group, p16-/p53+/HPV-, had a higher frequency of disease-specific deaths whether treated by radiation (XRT: 0% vs. 50%, p = 0.02) or surgery/XRT (15% vs. 36%, p > 0.05) controlling for stage. The p16-/p53+/HPV- patients also had worse recurrence-free survival compared to the referent group for radiation (0.0% versus 56%, p = 0.04, unadjusted) and for surgery/XRT (10% vs. 36%, p > 0.05, adjusted for stage) but not for other treatments. All other biomarker groups showed no difference in recurrence by treatment modality for all histologies combined or for treatment limited to SCC.

## Discussion

Although it is well-established that head and neck tumors detected with HPV oncoproteins, p16 overexpression, or p53 wild type, have better clinical outcomes, our study provides further insight into the molecular pathways of these tumor markers. The results show that there are different prognostic outcomes when these markers are examined together in tumors compared to outcomes based on single marker findings that one would not have predicted. This finding suggests that assessing multiple molecular markers in HNC patients at the time of diagnosis or treatment will better predict clinical outcomes and potentially the type of treatment targeted to those markers to improve prognosis.

Furthermore, despite evaluations of HNC limited to SCC, clinical outcomes were heterogeneous, suggesting that molecular characteristics are more important for prognosis. In our large clinical population followed for almost a decade, we have shown that HNC patients who have the combined tumor markers which individually are associated with better prognosis, p16+, p53-, and HPV-HR, also have the best clinical outcomes whereas those with the opposite status markers, p16-, p53+, and HPV-, have the worst disease-specific and recurrence-free survival.

In contrast, the other six joint biomarker groups had clinical outcomes different from each other that were not predictable based on the individual biomarker results. Distinctions in survival in these joint groups, compared to the single markers, remained after the hazard ratios for the individual markers were adjusted for the other two biomarkers. What this suggests is that the difference in survival outcomes may be due to interactions among the various molecular markers which multivariate analyses cannot detect by simple adjustment for confounding. Because of limitations in sample size in some of the joint groups, this hypothesis is still not altogether clear from our data and needs further investigation. The most distinctive finding was that no single marker in the joint effects groups was consistently pivotal for predicting worse overall, disease-specific, and recurrence-free survival hazard ratios. Although most biomarker groups had higher disease-specific mortality than the referent, among the worst disease-specific HRs were two joint groups that included p16-/p53+ regardless of their HPV status (p16-/p53+/HPV-HR and p16-/p53+/HPV-). These two groups also were among the lowest in 2-year median survival. Two other joint groups had similar, elevated recurrence-free HRs compared to the referent, despite having the exact opposite joint marker profile from each other (p16-/p53-/HPV-, HR = 8.6; and p16+/p53+/HPV-HR, HR = 9.8). In yet another contrast, despite overexpression of p53 in those with p16+/p53+/HPV-HR tumors, disease-specific survival was similar to the referent group which is p53-; yet the p16+/p53+/HPV-HR group had a 10-fold higher risk of recurrence compared to the referent. The evidence also indicated that compared to the referent joint group, the adjusted hazard ratios for recurrence-free survival were significantly worse or elevated in all other biomarker groups (HRs = 5.5-19.7). Because of the small sample size in some groups, these findings need additional numbers of patients to confirm our conclusions. This need for larger numbers of samples will be difficult to achieve because to date, few large studies have examined multiple biomarkers.

The large number of patients in this study enabled us to assess joint biomarkers and clinical outcomes while controlling for other risk factors associated with survival. We found strong evidence for alcohol and tobacco use to be associated with cases who were detected with HPV-HR tumors whether examined as individual or as joint markers, in contrast to some studies that believe that HPV and alcohol/tobacco risk factors characterize two distinct HNC tumor groups [20,21].

Two previous Italian studies [19,22] evaluated a small group of oropharyngeal carcinomas for p16, p53, and HPV but did not perform multivariate statistical analyses to control for confounding or examine survival for multiple gene interaction effects. A recent study in the U.S. [23] with limited sample size and several in Europe [24-26] also examined multiple markers and evaluated only OP survival. Not all cases were assessed for all tumor markers as done in this study and survival analyses in those and other studies were limited to p53/HPV or p16/HPV groups [6,11,16]. Furthermore, we included all major head and neck sites (oropharynx, oral cavity, and larynx/hypopharynx) since analyses showed no differences in joint marker outcomes by tumor site. Only 70% of our cases were diagnosed in stages III/IV compared to over 91% in the Italian study of patients and a much smaller portion of our HNC cases received surgery and radiation (35% versus 64%) [19,22]. These patient differences may account for variations shown in joint molecular profiles and prognostic outcomes between our studies. In contrast to Licitra et al., [19] we did not find that HPV-HR tumors were a distinct molecular group after evaluating a more complete molecular profile of the patient malignancies.

## Conclusions

Our findings highlight the complexity and importance of understanding the molecular pathways in tumor progression and the importance of characterizing the combination of these markers to more precisely identify clinical outcomes. Subgroup analyses by site need to be evaluated to be useful for clinicians to interpret. This will require larger numbers of patients defined by site, subsite, stage, and histology as well as by biomarker status to improve the application of these joint biomarker groups for clinical use.

## Methods

### Patients

The study population consisted of 237 histologically confirmed HNC patients enrolled between 1994 to 2004 at the University of Iowa Hospitals and Clinics for whom p16 and p53 immunohistostaining (IHC) and HPV detection had been performed at the time of analyses. Patients signed a human subjects consent form and completed a self-administered questionnaire regarding demographic characteristics and high risk HNC cancer behaviors (i.e. smoking, alcohol use, sexual practices), and a medical history including HPV-related diseases and lesions. Pathology records included prior cancer history, histology, stage, grade, nodal involvement, treatment, and outcome.

### Laboratory Methods

Formalin-fixed, paraffin-embedded tumor blocks were obtained to corroborate malignancy and tumor grade based on prepared hematoxylin and eosin-stained (H&E) slides. The tumor block with the highest percentage of malignant cells was used for HPV DNA detection and p16, p53 IHC. Tumor staging was based on the 1997 American Joint Committee on Cancer (AJCC) criteria [24] and grade, histology, and tumor site were obtained from the pathology report. Most cases were squamous cell carcinoma (SCC) with about 9% carcinomas of other carcinoma types. Because nonSCC histologies have not been routinely evaluated in HNC tumors and because of the established association between adenocarcinoma of the cervix and HPV-HR, we evaluated the molecular markers in these cases as well. Tumor sites were categorized as oral cavity, oropharynx, and larynx/hypopharynx (excluding nasopharynx) as recommended in the AJCC Cancer Staging Manual [27].

Laser capture microdissection (LCM) was performed to verify that HPV DNA detected in the same cut series was indeed present in the cancer cells and not in the adjacent normal epithelium when tissue specimens had less than 10% of tumor tissue or when they were initially found to contain an HPV nononcogenic, low-risk type as described previously [3]. If the HPV or β-globin results from block tissues or LCM were divergent, the LCM findings were used in the analysis because of its higher sensitivity and accuracy in targeting tumor cells. The Qiagen DNA Tissue Kit protocol was used in the DNA extraction. HPV DNA was detected by PCR with MYO9/11 primers and dot blot hybridization. Dot blot positive samples were amplified by with primers of GP5+/GP6+ or GP5+/MY09 and typed by automated sequencing resulting in all types identified through the BLAST system. Detailed procedures have been described elsewhere [3]. HPV- specimens are defined as those that are negative for HPV-HR types; HPV low-risk types are included in the HPV- group because they are nononcogenic and not associated with an increased risk of HNC [3,16].

P16 and p53 IHC was performed as described previously [7]. Standard positive and negative p16 and p53 slides were used as controls in their respective assays. Then tissue sections were counter-stained with hematoxylin, dehydrated in graded alcohol, cleared in xylene, and mounted. All IHC slides were reviewed by one pathologist (THH) and strong nuclear and cytoplasmic staining defined a positive reaction. The p16 and p53 staining was scored on a semi-quantitative scale and then dichotomized to positive and negative for statistical analyses. The initial scale for both biomarkers was as follows: 1 = negative for staining, 2 = <10% positive staining, 3 = 10%-80% positive, 4 = ≥80%<100% positive; and 5 = 100% positive in every tumor cell. These categories were collapsed with category 1 defined as negative, and 2, 3, 4, or 5 defined as positive. There were only 2 cases in category = 2 of p16+ and 4 cases of p53+; category 3 included 7 p16+ and 18 p53+ cases. The numbers in these two categories did not alter the statistical findings when included in the analyses of this large study. Only stained tumor cells with a positive result were defined as positive and nontumor cells such as multinucleated giant cells and epithelia were not included.

### Statistical Methods

Risk factors examined in the analyses were age, gender, alcohol, tobacco, tumor site, grade, stage, and treatment. Logistic regression was used to calculate the adjusted significance of associations between HPV, p16 and p53 and demographic characteristics and risk factors. Results for SCC and nonSCC were compared for differences in biomarker frequencies and in adjusted multivariate statistical analyses. Except for the few significant differences between these two groups, results are reported for the combined histologies in the analyses. Statistical comparisons in median survival were analyzed using the median Wilcoxon test. Survival curves were estimated by the Kaplan-Meier method and differences between groups were assessed using the log-rank test. Hazard ratios (HRs) were estimated using the Cox proportional hazards model. Proportional hazards and other model-building assumptions were assessed. Overall and disease-specific survival, and recurrence were measured in years from the date of diagnosis until death (or recurrence) or until the patient was last known to be alive. Disease-specific survival was based on events related to deaths from HNC. Patients with an indeterminate cause of death were excluded from the overall survival analysis. Date of death or date last known to be alive was obtained from the NCI Iowa SEER Cancer Registry [28], university hospital tumor registry, and National Death Index [29]. All independent variables were assessed for multiplicative interactions in the models and the joint biomarker groups were assessed for additive interactions with the synergy index^10^. Statistical results were based on two-tailed tests with statistical significance at p < 0.05. Statistical analyses were performed using SAS 9.1.

## Abbreviations

HR: hazard ratios; CI: confidence intervals; HPV-HR: human papillomavirus high-risk; OR: odds ratios; HNC: head and neck cancer; IHC: immunohistostaining; SCC: squamous cell carcinoma; XRT: radiation treatment.

## Competing interests

"The authors declare that they have no competing interests." That includes financial and non-financial competing interests on the part of any of the authors.

## Authors' contributions

EMS contributed to the conception and design, acquisition of data, and interpretation of data, drafted and revised the manuscript. LMR contributed to the statistical analyses, interpretation of data, drafted and revised the manuscript. HH contributed to the interpretation of the clinical data, drafted and revised the manuscript. THH contributed to oversight and interpretation of the laboratory analyses, drafted and revised the manuscript. LPT contributed to the interpretation of the molecular pathology and biology aspects, drafted and revised the manuscript.

All authors read and approved the final manuscript.
